# Item Response Theory Investigation of Misophonia Auditory Triggers

**DOI:** 10.3390/audiolres11040051

**Published:** 2021-10-14

**Authors:** Silia Vitoratou, Nora Uglik-Marucha, Chloe Hayes, Mercede Erfanian, Oliver Pearson, Jane Gregory

**Affiliations:** 1 Psychometrics and Measurement Lab, Biostatistics and Health Informatics Department, Institute of Psychiatry, Psychology and Neuroscience, King’s College London, London SE5 8AB, UK; silia.vitoratou@kcl.ac.uk (S.V.); eleonora.uglik-marucha@kcl.ac.uk (N.U.-M.); chloe.1.hayes@kcl.ac.uk (C.H.); oliver.pearson@kcl.ac.uk (O.P.); 2 UCL Institute for Environmental Design and Engineering, The Bartlett, University College London, London WC1H 0NN, UK; mercede.erfanianghasab.18@ucl.ac.uk; 3 Centre for Anxiety Disorders and Trauma, South London and Maudsley NHS Foundation Trust, London SE5 8AZ, UK; 4 Department of Experimental Psychology, University of Oxford, Oxford OX2 6GG, UK

**Keywords:** misophonia, psychometrics, item response theory, sound sensitivity, S-Five

## Abstract

Misophonia is characterised by a low tolerance for day-to-day sounds, causing intense negative affect. This study conducts an in-depth investigation of 35 misophonia triggers. A sample of 613 individuals who identify as experiencing misophonia and 202 individuals from the general population completed self-report measures. Using contemporary psychometric methods, we studied the triggers in terms of internal consistency, stability in time, precision, severity, discrimination ability, and information. Three dimensions of sensitivity were identified, namely, to eating sounds, to nose/throat sounds, and to general environmental sounds. The most informative and discriminative triggers belonged to the eating sounds. Participants identifying with having misophonia had also significantly increased odds to endorse eating sounds as auditory triggers than others. This study highlights the central role of eating sounds in this phenomenon and finds that different triggers are endorsed by those with more severe sound sensitivities than those with low sensitivity.

## 1. Introduction

Misophonia is best characterised by a disproportionate emotional response occurring in association with a decreased tolerance for certain sounds [1,2,3,4,5,6]. The auditory “triggers” most reported are oral sounds, such as the sound of others eating [2,7,8], nasal/breathing sounds [7,8] and repetitive sounds including repetitive tapping or rustling sounds made by humans (e.g., finger tapping) or machines (e.g., clock ticking) [2,9].

The emotional responses to these trigger sounds include anger, irritation, disgust [7,10,11] and anxiety [2,10,12]. In addition to the emotional response, individuals sometimes report physical tension building in the chest, neck or other parts of the body [10,13].

The epidemiology of misophonia is far from understood. Two studies reported on the incidence of misophonia, ranging from 6 to 20% in student samples with different ethnic backgrounds [12,14]. The age of onset of misophonia symptoms has been reported as typically between 10 and 13 years old by several studies [2,3,4,10,11,15]. The diagnostic criteria proposed by Schröder et al. [11] and revised by Jager et al. [7] categorised it as a discrete psychiatric disorder, though it has also been reported alongside audiological complaints [6,16] and broader sensory intolerance [17]. Further exploration of this phenomenon is required before categorisation and diagnostic criteria can be determined [18]. In the meantime, improved means of detecting the phenomenon are needed.

The impact of misophonia can range from mildly hindering to highly debilitating [2]. Clinical misophonia samples report higher levels of disability than non-clinical samples [12,14] and individuals report that misophonia impacts negatively on work and relationships [7,19,20], and can lead to severe distress and suicidal thoughts [10].

Characteristic of misophonia is the disproportionate, intense and negative reaction to day-to-day sounds which non-sufferers find easy to tune out or only mildly aversive [2]. The auditory triggers tend to be pattern-based and repetitive, with either organic (e.g., chewing) or non-organic (e.g., clock ticking) origins [1,2,4,12,21]. One large study found that all participants reported an emotional reaction to at least one sound of oral or nasal origin, and the researchers proposed that a reaction to one of these types of sounds should be required in order to diagnose the condition [7]. Another study [9] employing machine-learning algorithms, found that those with misophonia rated all three sound categories (oral/nasal, human-produced non-oral/nasal and non-human/nature sounds) as more aversive than controls did, and that both the misophonic and control groups rated the oral/nasal sounds as causing more discomfort than the other two types of sounds (i.e., human-produced non-oral/nasal sounds and non-human/nature sounds). They reported that the inclusion of all three types of sounds improved predictions of severity and classification of misophonia and proposed that misophonia research should not be confined to using only human-produced oral and nasal sounds.

In the present study we seek to examine individual misophonic triggers in greater depth using psychometric tools, such as factor analysis and item response theory (IRT). Our primary goals were to identify: (i) which triggers are more discriminative between people with different levels of misophonic experience or sound sensitivity; (ii) which triggers indicate higher sound sensitivity (more severe); (iii) which triggers are more informative (reliable, precise) for high, average and low sensitivity. In addition, we explore which triggers can be considered more misophonia-specific, comparing pilot data from people who identify as having misophonia and those who report that they do not have misophonia.

## 2. Materials and Methods

### 2.1. Sample

Data were collected from two populations as part of the Selective Sound Sensitivity Syndrome Study [22] during the second sampling wave. The first sample, hereafter referred to as the misophonia specific sample (MS), came from a call in misophonia support groups on social media (Facebook, and Reddit). The second sample, hereafter the misophonia non-specific sample (MNS), came from a general call in social media by the authors (using both personal and professional handles). Inclusion criteria were being at least 18 years old and fluent in English. Having a severe learning disability was the only exclusion criterion. All participants completed online forms after reading an information sheet about the study and giving consent (ethics approval reference RESCM-19/20-11826).

### 2.2. Measures

This study was part of a larger study validating a new tool for measuring misophonia, the selective sound sensitivity syndrome scale (S-Five) [22] and included two more tools to assess misophonia, the Misophonia Questionnaire (MQ) [12] the Amsterdam Misophonia Scale (A-MISO-S) [11]. For the purposes of the present study, participants were asked to rate the intensity of their reaction to 35 potential misophonia trigger sounds (“triggers”), using a five-point ordinal scale (0: does not bother me, 1: temporarily distracting, 2: very distracting, 3: distressing and 4: unbearable). An individual’s frequency and intensity trigger score (FITS) was created from the sum of responses to these 35 items. A responder was considered to endorse a particular trigger if they rated that item as distressing or unbearable (i.e., a rating of 3 or 4) and binary items were created (1: trigger endorsed and 0: trigger not endorsed). The number of triggers endorsed by an individual formed their trigger endorsement score (TES).

To establish whether someone self-identified as having misophonia, we asked “Do you identify as having misophonia?”, with the option of yes, no or unsure. Participants were also asked if they had been given a formal diagnosis of tinnitus, hyperacusis or any of a range of common mental health problems (for example, depression or generalised anxiety disorder).

### 2.3. Factor Analysis

Item factor analysis for categorical items (IFA) was conducted using the weighted least squares estimator (WLSMV) [23] in MPlus [24] using Promax rotation. The number of items to be retained was decided taking under consideration the Guttman–Kaiser criterion [25,26], scree plot [27] and the percentage of variance explained [28]. Measures of absolute and relative fit are also reported, namely the root mean square error of approximation (RMSEA): values below 0.05 indicate close fit [29], the relative chi-square Rel χ^2^: values close to 2 indicate adequate fit [30], the comparative fit index (CFI): values above 0.90 indicate close fit [31], the Taylor-Lewis (TLI): values above 0.90 are required for close fit [32], and the standardised root mean residual (SRMR): values below 0.08 suggest good fit [29].

### 2.4. Item Response Theory

The two-parameter item response theory model (2PL-IRT) [33], was used to evaluate the severity (difficulty), discrimination ability and information (precision) of trigger endorsement. Severity refers to the amount of sound sensitivity required for a person to endorse a trigger (for example, how sensitive to sounds a person must be to be distressed, say, by the sound of someone whistling). Discrimination ability refers to the ability of a trigger to tell apart people with different levels of sensitivity (for example, how well can we discriminate those with high sensitivity from those with low, based on whether they are distressed by whistling or not). The item information refers to how reliable (precise) a trigger is as an indicator of the sound sensitivity (for example, endorsing whistling as a trigger may be an informative indicator of the sensitivity for those with low sensitivity but not informative for those with high sensitivity, that is, whistling can be precise for low scorers but not for high scorers).

### 2.5. Reliability

Internal consistency was evaluated using Cronbach’s [34] alpha coefficient, the item-total correlations and the computation of the alpha if the item was omitted. Stability (test-retest reliability assessed at two weeks) was evaluated via Cohen’s [35] weighted Kappa (k_w_) for each item, following Landis and Koch [36] interpretations, along with the percentage of agreement and the Psi coefficient [37]. Precision of the measurement per trigger was also evaluated via the item response theory information.

### 2.6. Hypothesis Testing

Logistic regression was used to study the odds of endorsing a trigger in relation to reporting identifying with misophonia. To adjust for multiple comparisons over the 35 triggers, we used the Benjamini and Hochberg [38] method. One-way ANOVAs were used for differences in the means between groups and Pearson’s chi-square was used to identify associations between categorical variables. These analyses were conducted in R [39].

## 3. Results

### 3.1. Sample: Demographic and Clinical Characteristics

The mean age of the misophonia specific sample (MS; N = 613) was 36.4 years (sd = 13.4, median = 34, min = 18 and max = 75 years). With respect to gender, 78.2% identified as “female”, 18.7% as “male” and 3.1% as “other” (non-binary or other). Most of the participants selected the “White/Caucasian” ethnicity (92%; Hispanic or Latino 2%; Black 1%; and missing 5%).

The mean age of the misophonia non-specific sample (MNS; N = 202) was 33.8 years (sd = 11.5, median = 31, min = 18 and max = 71 years). With respect to gender, 77.2% identified as “female”, 16.8% as “male” and 4.5% as “other” (non-binary or other). Most of the participants reported being of “White/Caucasian” ethnicity (83%, mixed 7%, Asian 5%, Hispanic 3%, Black 1% and other or missing 1%).

All participants in the MS stated that they identified as having misophonia. In the MNS, 106 (52.5%) individuals stated that they identified as having misophonia, 54 (26.7%) stated that they do not have misophonia, and 42 (20.8%) said they were unsure if they had misophonia. A formal diagnosis of depression was reported by 37% of participants in the MS, and 32.2% in the MNS. Generalised anxiety disorder was reported by the 25.6% and 25.2% of MS and MNS, respectively. Tinnitus was reported by 10.3% and 9.4% of MS and MNS, respectively. 

### 3.2. Trigger Psychometric Properties-Misophonia Specific Population

#### 3.2.1. Intensity of Misophonic Triggers

Table 1 presents the descriptive indices of the trigger items for the misophonia specific sample. The items with the highest intensity ratings (thus higher means) were “I2 eating with open mouth”, “I23 chewing gum”, “I6 smacking lips”, “I7 slurping” and “I3 crunching”.

#### 3.2.2. Endorsement of Misophonic Triggers

The following analyses use the binary “trigger endorsement” item described in the Measures section (0: trigger not endorsed and 1: trigger endorsed).

##### Factor Analysis

**Figure 1:**
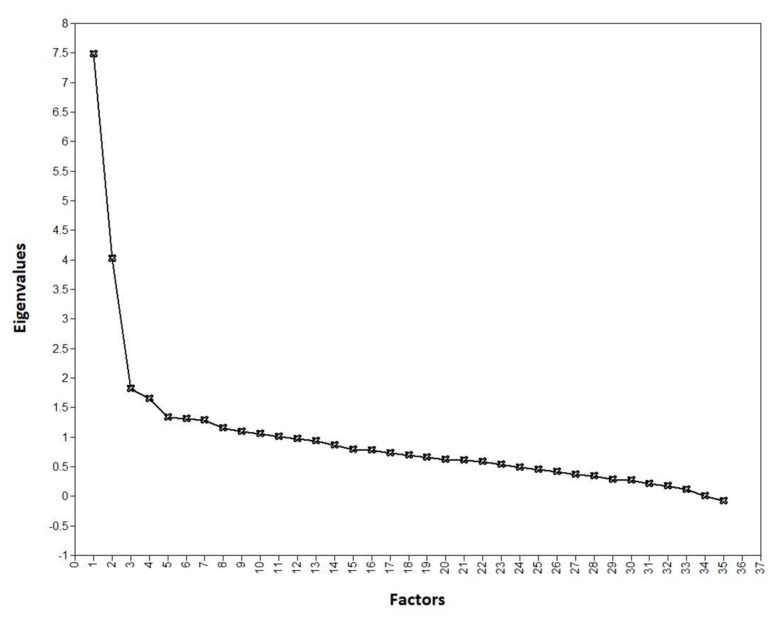
Scree plot.

The first step in our analysis was to identify the underlying dimensions of the set of triggers, to be able to conduct the rest of the analysis within dimension. Eleven eigenvalues above 1 were present in the sample covariance matrix (7.48, 4.02, 1.82, 1.65, 1.33, 1.31, 1.28, 1.16, 1.09, 1.05 and 1.01), suggesting up to 11 factors according to Guttman–Kaiser rule. However, the goodness of fit indices indicate that close fit is achieved for the 3-factor solution (rel χ^2^ = 1.36, RMSEA = 0.024 95 CI: (0.020, 0.029), CFI = 0.96, TLI = 0.95 and SRMR = 0.064), which is also supported by the scree plot (Figure 1). In terms of content, the triggers loaded into three coherent dimensions, namely eating sounds (eating with mouth open, normal eating, mushy foods, lip smacking, slurping, swallowing, crunching food and chewing gum), nose/throat sounds (repetitive sniffing, repetitive coughing, blocked nose breathing, normal breathing, throat clearing, hiccups and snoring) and environment sounds (low frequency bass sounds, whistling, car engine, certain letter, certain words, certain accents, humming object, rustling plastic or paper, tapping, clock ticking, keyboard tapping, footsteps, muffled sounds, cutting nails or skin, joint, sneezing, kissing, cutlery, baby crying and repetitive barking). Increasing the number of factors resulted essentially to the same three themes and dimensions consisting of cross-loadings, rather than standalone factors. We therefore accepted the three-factor solution, and the item loadings are presented in Table 2.

##### Reliability

With respect to test-retest reliability, all triggering sounds demonstrated satisfactory stability in time according to all coefficients (Table 2). Cronbach’s alpha was moderately high (eating sounds a = 0.77 and general environment sounds a = 0.74) to moderate (nose/throat dimension a = 0.56). As endorsement of one trigger does not theoretically imply endorsement of another trigger of the same family, these values were considered satisfactory. According to alpha-if-item-deleted and to the item-total correlations, none of the items were problematic (Table 2). Evidence towards the reliability (precision in measurement) for each trigger separately is presented in the next section, via the IRT information.

##### Item Response Theory

**Figure 2:**
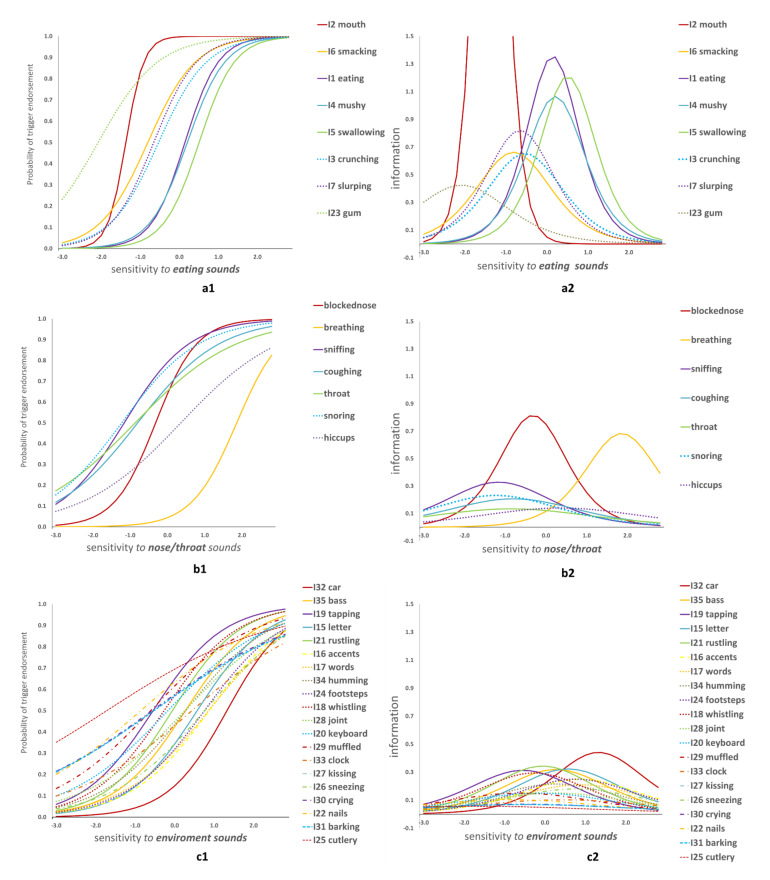
Item characteristic curves (ICC) and item information curves (IFC) per dimension.

The two-parameter logistic model was fitted for the endorsement of the triggers (separately within each dimension) and the estimated parameters are presented in Table 3. The severity, discrimination ability, and the item information are shown graphically in Figure 2.

Among the eating sounds (Figure 2(a1)), the most discriminating trigger was “listening to people eating with their mouth open” (I2). The least discriminating trigger was “listening to people chewing gum loudly” (I23). That is, among all triggers, I2 corresponds to the larger differences in sound sensitivity between those who endorse it and those who do not. On the contrary, I23 endorsement corresponds to the least notable sensitivity differences. Chewing gum was also the least severe symptom in this dimension of triggers (i.e., it was endorsed even by those with low sensitivity to sounds). The trigger indicating the most severe sensitivity was “swallowing sounds” (I5). Interesting results occurred related to the information provided by the eating triggers. The eight items were divided in three groups: one most informative (more reliable, precise) for low scorers, one most informative for average sensitivity and one most informative for high scorers (Figure 2(a2)). For example, “eating with the mouth open” (I2) was very informative for people with low sensitivity but not informative otherwise. “Eating” (I1), “mushy” (I4) and “swallowing” (I5) were very informative for high scorers but less informative otherwise. The rest of the items performed very well for average scorers.

Among the nose/throat sounds (Figure 2(b1)), the most discriminating triggers were “listening to people normally breathing” (I8) or “breathing through a blocked nose” (I9). The rest of the items had similar discrimination parameters with one another, of low magnitude. The most severe symptom was being triggered by someone’s “breathing” (I8) and the least severe were “sniffing” (I12) and “snoring” (I14). In terms of precision (Figure 2(b2)), the “blocked nose” sound was highly informative for the average person and for low scorers (down to -2 standard deviations from the mean). Moderate information was also provided by the rest of the sounds, for low scorers as well. Being triggered by others’ “breathing” (I8) was highly informative for high scorers, with a peak at 2 standard deviations above the mean.

The large cluster of general environment sounds (Figure 2(c1,c2)) appears to be divided in two groups of triggers. The first group consists of the triggers with low discrimination ability (“crying” I30, “nails” I22, “cutlery” I25, “barking” I31, “kissing” I27 and “clock” Ι33) and low information at all ranges of the sensitivity. The second group consisted of the rest of the sounds, which were more discriminative and informative (around the average person) with comparable estimated values. The only exception is the “car engine” (I32) which appears to be the most discriminative, severe and informative trigger, with a peak of precision towards the high scorers (about 1.5 standard deviations above the average person).

### 3.3. Hypothesis Testing—Misophonia Non-Specific Sample MNS

The next step in our analysis was to test for differences in the odds of endorsing triggers in the MNS, in relation to a person identifying as having misophonia, not having misophonia or being unsure whether they have misophonia.

FITS was statistically different across all three groups (pairwise comparisons *p* < 0.5, Bonferroni adjusted), with higher scores in those self-identifying as misophonic (FITS mean = 60.1, sd = 20.2), followed by those who were unsure whether they had misophonia (FITS mean = 42.9, sd = 21.7) and those who do not identify as having misophonia (FITS mean = 32, sd = 14.6). The number of triggers endorsed by those who reported misophonia (TES mean = 18.5, sd = 5.5) was significantly higher than those who were unsure (TES mean = 13.5, sd = 4.3, *p* < 0.001), and those who reported that they do not have misophonia (TES mean = 11.8, sd = 5, *p* < 0.001). The latter two groups did not differ significantly in the number of triggers endorsed (*p* = 0.302).

Subsequent analyses included only those who stated that they identify as having misophonia (M^+^; n = 106) and those who stated that they did not have misophonia (M^−^; N = 54). The responses of people who stated that they are unsure were omitted from these analyses.

The two groups did not differ in terms of *gender* (M^−^ vs. M^+^ percentage of females: 84.6% vs. 84.8%, χ^2^ = 0.001, df = 1, *p* = 0.970). Statistically significant differences occurred with respect to reported *depression diagnosis* (M^−^ vs. M^+^ percentage of positives: 14.8% vs. 36.8%, χ^2^ = 8.329, df = 1, *p* = 0.004), reported *anxiety diagnosis* (M^−^ vs. M^+^ percentage of positives: 13% vs. 27.4%, χ^2^ = 4.252, df = 1, *p* = 0.039) and *age* (M^−^ mean = 32.4, sd = 8.4 vs. M^+^ mean = 36.25, sd = 12.8; t = 2.262, df = 148.7, *p* = 0.025).

A logistic regression model was subsequently fitted for each trigger separately (dependent variable), to investigate the odds of endorsement in relation to misophonia (1: reported present, 0: reported absent), adjusted for age (years), depression and generalised anxiety diagnoses (1: diagnosis reported, 0: no diagnosis reported, in both cases), sex (1: male, 0: female) and age (years). Table 3 presents the odds ratio (OR) for each trigger.

The participants in M^+^ were overwhelmingly more likely to report that they found the eating sounds distressing or unbearable compared to those that do not report misophonia (ORs varied from 43.6 to 15.1, *p* < 0.001 in all cases, see Table 3). For example, people with misophonia were over 40 times more likely to endorse as a trigger normal “eating” (I1) and “chewing with mouth open” (I2). In the case of “crunching” (I3), the adjusted (for other covariates) odds ratio could not be computed as only one person from M^+^ group did not endorse the trigger (endorsement M^+^ 98.6% vs. M^−^ 1.4%, chi-square = 61.314, df = 1, *p* < 0.001).

With respect to nose/throat related sounds, the odds of endorsing “blocked nose breathing” (I9) as a trigger were 22 times higher in the misophonia group and “repetitive sniffing” (I12) was over 3 times higher. No other trigger of this family was different between people from M^+^ and M^−^, although the odds ratio could not be computed also in the case of normal breathing (I8) since no individual from the M^−^ sample endorsed it (endorsement M^+^ 10.4% vs. M^−^ 0%, chi-square = 6.017, df = 1, *p* = 0.014). For the general environmental sounds, the largest difference occurred in cutlery sounds, where people from M^+^ were 3.5 times more likely to endorse this trigger. Other triggers whose odds of endorsement was higher in the M^+^ sample were rusting paper or plastic, whistling sound, keyboard tapping and cutting nails (ORs varied from 3 to 2.6, see Table 3).

## 4. Discussion

This study aimed to shed light on the most frequently reported triggers by those who identify as experiencing misophonia. Using contemporary psychometric methods, we studied the properties of the triggers in terms of internal consistency, stability in time, precision, severity, discrimination ability and information. We also compared participants with and without misophonia in terms of the intensity of their response to triggers and the number of sounds they endorsed as triggers.

Factor analysis indicated that the triggers clustered into three dimensions of sound sensitivity, namely sensitivity to eating sounds, nose/throat sounds and general environmental sounds. These groups were moderately internally consistent, indicating that people who are triggered by one sound in a group are somewhat likely to be triggered by other similar sounds, and the reported sensitivities were stable over a two-week period. Our results complement previous work suggesting oral and nasal sounds are the predominant misophonic triggers [2,7,12]. For instance, Jager et al. [7] reported that 96% of their sufferers were triggered by eating-related sounds and 85% by nasal sounds, with frequent complaints about repetitive environmental sounds, see also [12,18]. The present study adds to previous work by identifying categories of sounds, demonstrating that the presence of a particular trigger may increase the probability of endorsing another trigger of a similar kind. It was interesting to note that eating sounds formed a factor distinct from nose/throat sounds, while previous research has combined these into a single oral/nasal category [9]. Human-produced repetitive sounds (not related to eating or nose/throat, e.g., footsteps) clustered together with non-human repetitive sounds (e.g., clock ticking), where these had previously been grouped separately [9]. This categorisation of sounds will be particularly beneficial for future experimental research where repeat trials of similar sounds are required, such as neuroimaging studies. Future research could also explore similarities and difference within and between the categories of sounds in relation to emotional and behavioural responses or impact on functioning.

IRT analysis identified several triggers that are indicative of higher sound sensitivity (items with higher difficulty or severity parameter in IRT terms). Higher levels of sound sensitivity were required for one to be triggered by other people’s swallowing, breathing (normal), accents and pronunciations and car engines. At the other end, the sounds endorsed by those with mild sound sensitivities included chewing gum, throat clearing and cutlery noises.

The discrimination ability of the triggers was also investigated using the IRT model, that is, the ability for trigger endorsement to distinguish between people with different levels of sound sensitivity. The most discriminative sound was the eating sound, which is at the very core of misophonia, having three- and four-times higher discrimination parameter than all other sounds. This core element is substantially supported by previous work regardless of the populations of the sufferers [2,4,7,11,12,15,40,41,42,43].

Finally, the IRT model provides us evidence of the reliability of the indicators (here trigger sounds) for specific levels of the latent variable under measurement (here sound sensitivity). Endorsing is a reliable indicator of the sensitivity for most of the triggers, but mainly for low and average scorers. For the high scorers, it appears fewer triggers provide highly reliable information. For instance, “lip-smacking” and “slurping” provide the peak of their information for individuals with sound sensitivity about one standard deviation below the mean. At this point, “swallowing” comes with low information. As we move to higher scorers though, “swallowing” becomes increasingly reliable, and is the most reliable indicator for individuals with sound sensitivity higher than average and closer to one standard deviation above average. Likewise, “normal breathing” became an increasingly reliable indicator in high scorers in sensitivity to nose/throat sounds. These results allow us for the first time to understand how the triggers manifest in different points of the sensitivity continuum. Considering that swallowing and normal breathing are typically quieter than the eating and nose/throat sounds endorsed by those with low sound sensitivities, it is possible that as someone becomes more sensitive, they simply detect and are bothered by sounds at a lower volume. If that were the case, then those with low sensitivities may be more bothered by swallowing and normal breathing if they were as loud as chewing sounds or throat clearing. Alternatively, there may be other acoustic or semantic properties in these two sounds that are not experienced as distressing by those with lower sound sensitivities. This could be tested in an experimental setting. Further research is also needed to better understand how things change as sensitivity increases.

The second part of our analysis focused on preliminary hypotheses testing in pilot data from a misophonia non-specific sample. Using these data, we verified that the people who identify as having misophonia report more triggers and with higher impact, than those who do not. This result suggests that self-identification with the condition is a reliable indication of the existence of the sound sensitivity and provides evidence of criterion validity for these two indices.

The odds of endorsing a trigger were computed in relation to identifying (or not) with misophonia, adjusted for age, gender and reported anxiety and/or depression disorders. In line with our other results, we identified that eating sounds are immensely more likely to trigger people who identify as having misophonia than those who do not, even after adjusting for other covariates. Compared to those without misophonia, those with misophonia were more than 40 times more likely to be triggered by “normal eating” or “eating with mouth open”, and more than 20 times more likely to be triggered by loud/unusual breathing sounds. This finding is consistent with the notion that breathing or nasal related sound tend to trigger misophonic responses, but less frequently relative to eating-related sounds [2,7,10,11,12]. Interestingly, of those who did not identify with having misophonia, no individual endorsed “normal breathing” as a trigger, but 10% of those with misophonia did.

Within the general environment sounds, clock ticking, baby crying, tapping and dog barking were not significantly more likely to be endorsed by those with misophonia than those without. These sounds have previously been reported as distressing to those with misophonia [18]. It is possible that being bothered by these sounds is not specifically related to having misophonia, but that in previous studies individuals had misattributed their reaction to these sounds as being part of their misophonia. Other environmental sounds, including cutlery, rustling and keyboard tapping were more likely to be endorsed by those identifying with misophonia. Jager et al. [7] also found that participants reported aversion to these types of sounds, but that they were only reported alongside oral and/or nasal trigger sounds. The research team proposed that misophonia should not be diagnosed in the absence of oral/nasal sounds, and that responding only to environmental sounds may indicate a more general sound sensitivity rather than misophonia, even if the response to these sounds was similar [7]. This raises an interesting question of how “selective” the sounds need to be for the label of misophonia to be applicable, and how much weight is placed on the nature of the reaction and the associated impairment rather than the specificity of the sound. Misophonia is typically reported as being an aversion to oral and nasal sounds, and thus it is possible that it is individuals bothered by those, particular, sounds who have been more likely to identify with the phenomenon and to participate in research and seek treatment. While oral and nasal sounds may be the predominant reported triggers, we need further research examining the specific emotions experienced as a result of each sound to improve our understanding of these other environmental triggers sounds in a way that could not be captured with the binary endorsement measure in the present study. This may help to establish whether the phenomenon of misophonia extends to these other sounds.

The present study provides further support for the notion that a defining feature of misophonia is the extreme aversion to eating sounds. It is not clear why eating sounds, in particular, should be such a key part of this phenomenon. Cox [44] proposed that a disgust reaction to sounds associated with bodily secretions and excretions, including eating sounds, may have evolved as a protective mechanism against high levels of pathogens found in bodily fluids. That is, a heightened sensitivity to eating sounds may have developed in some people as a means of avoiding contamination from potential pathogens in spit that could be spread when eating with an open mouth. Cox [44] also reported that the pattern of disgust reaction to sounds related to bodily fluids was disproportionate to what would be required purely for the purpose of disease avoidance, suggesting that this could be the result of the meaning applied to the behaviour, heightening the disgust reaction. As discussed in McKay and Acevedo [45], it is possible that misophonia begins with a disgust reaction to sounds related to bodily fluids, followed by a sense of moral disgust towards the person making the sounds, contributing to feelings of anger. The feelings of disgust and anger associated with a potential contaminant (e.g., spit from open mouth chewing) may then become paired with neutral sounds (e.g., other benign sounds made by the same person). The misophonic individual may feel ashamed of their disproportionate reaction [10] or anxious about having outbursts towards others [2], thereby heightening the intensity of the emotional reaction to these sounds over time.

In terms of why eating sounds are reported as more distressing than more obvious illness-related sounds (e.g., sniffing and breathing through a blocked nose), there are a few possible explanations. It could be that with illness sounds, the initial disgust reaction makes more immediate sense, and thus is easier to dismiss, compared with a reaction to eating sounds where the link with contamination is less obvious. The individual could then become preoccupied with their reaction, similar to someone with obsessive compulsive disorder questioning the meaning of an intrusive thought [46]. Another explanation is that the experience of sympathy towards someone who is sick helps mitigate the reaction to the sounds, or that the sense of moral disgust is reduced when the person is seen as not to blame for the sounds. Additionally, eating involves repetitive jaw movement, and misophonic individuals have reported that reactions intensify when a sound is paired with a visual stimulus [7]. This phenomenon is supported by recent research finding increased activation of the orofacial motor area in individuals with misophonia when hearing a trigger sound, proposed to be a “mirroring” of the person making the sound [47]. To shed light on the disproportionate response to eating sounds in particular, further research on is needed on the cognitive aspects of the reaction, the acoustic properties of the sounds and the corresponding neurophysiological responses.

There were limitations to our study. As yet, there are no formally agreed diagnostic criteria for misophonia, and we relied on self-report of the condition. Therefore, these results reflect the experience of individuals who are familiar with the term misophonia and identify with having the condition. The “no misophonia” comparison group was created based on the individual stating that they did not have misophonia, with the assumption that they are familiar with the term and believe they do not have misophonia (with the “unsure” category assumed to include those unfamiliar with the term as well as those familiar but not sure if they have it). It cannot be considered a true clinical and non-clinical comparison, although the results serve as preliminary results that could be examined in further studies using diagnostic interviews or misophonia psychometric tools [22,48]. Future studies would also benefit from using samples representative of the general population, rather than recruited via social media, as in the present study. Future research should also include comparisons according to gender and ethnicity. It would also be helpful to conduct research using structured clinical interviews to establish whether self-identification with misophonia is consistent with a formal clinical diagnosis.

Another limitation was the use of a binary measure of trigger endorsement. For the purposes of this study, we considered a trigger to be endorsed if it was reported as causing distress, but not it if was reported as “very distracting”. It is possible that this choice for the binary measure could have excluded some triggers that cause significant impairment as a result of distraction, but which were not experienced as distressing to the individual. Future research would benefit from gathering more specific information about the reactions to trigger sounds and an exploration of primary, secondary and anticipatory emotions.

We also did not ask about the source of the trigger sounds. There is some evidence that reactions to trigger sounds may varying depending on who is making the sound [2,10], and this would be useful to explore further, to establish whether individuals who have stronger reactions to, say, a particular family member, also show broader traits of sensitivity to certain sounds. Additionally, it would be useful to look at these auditory sensitivities in the context of more general sensory sensitivities, and to compare with other groups showing high levels of sensory sensitivity, such as those with autism spectrum conditions [49]. Finally, only auditory triggers were explored. Future research would benefit from including repetitive visual triggers (e.g., leg shaking) and possibly combined audio and visual triggers (e.g., the sound of eating paired with jaw movement).

## 5. Conclusions

This study sheds new light on the triggers sounds most frequently reported by those with misophonia. The categorising of sounds into eating, nose/throat and environmental sounds through factor analysis has provided a useful framework for future research. Future research could include making comparisons between these groups of sounds in terms of acoustic and semantic properties, and the physiological, emotional and neurological responses to these sounds. The detailed information provided by item response theory may support the development of simple screening tools for misophonia based on the triggers endorsed. This study contributes to the growing base of research into misophonia and highlights the importance of well-designed control studies and experimental research to improve understanding of this complex phenomenon.

## Figures and Tables

**Table 1:** Item descriptive indices in misophonia specific sample (time 1: N = 613) and test-retest indices (N = 42)—intensity items.

Sound	Mean (sd)	Median (Q1–Q3)	Agreement in Time	Weighted Cohen’s k	Psi (95% CI)
I1 Normal eating	2.2 (1.2)	2 (1–3)	0.96	0.81	0.86 (0.82, 1)
I2 Chewing/Mouth open	3.5 (0.8)	4 (3–4)	0.98	0.82	0.79 (0.74, 1)
I3 Crunching	2.7 (1.3)	3 (2–4)	0.97	0.91	0.89 (0.86, 1)
I4 Mushy foods	2.0 (1.4)	2 (1–3)	0.92	0.70	0.83 (0.78, 1)
I5 Swallowing	1.8 (1.3)	2 (1–3)	0.97	0.86	0.87 (0.83, 1)
I6 Lip smacking	2.9 (1.1)	3 (2–4)	0.94	0.71	0.84 (0.79, 1)
I7 Slurping	2.8 (1.2)	3 (2–4)	0.97	0.88	0.87 (0.83, 1)
I8 Normal breathing	0.9 (1.1)	1 (0–1)	0.96	0.57	0.83 (0.79, 1)
I9 Loud/unusual breathing (blocked nose)	2.6 (1.2)	3 (2–4)	0.96	0.80	0.84 (0.80, 1)
I10 Throat clearing	1.9 (1.3)	2 (1–3)	0.94	0.63	0.83 (0.77, 1)
I11 Repetitive coughing	2.1 (1.2)	2 (1–3)	0.95	0.78	0.85 (0.81, 1)
I12 Repetitive sniffing	2.6 (1.2)	3 (2–4)	0.98	0.83	0.88 (0.84, 1)
I13 Hiccups	0.9 (1)	1 (0–1)	0.94	0.52	0.79 (0.74, 1)
I14 Snoring	2.6 (1.4)	3 (2–4)	0.96	0.85	0.87 (0.83, 1)
I15 Certain letter sounds	1.0 (1.3)	0 (0–2)	0.98	0.92	0.82 (0.77, 1)
I16 Certain accents	0.8 (1.2)	0 (0–1)	0.97	0.76	0.75 (0.70, 1)
I17 Certain words	0.9 (1.2)	0 (0–2)	0.96	0.66	0.77 (0.71, 1)
I18 Whistling sound	1.8 (1.4)	2 (1–3)	0.94	0.80	0.85 (0.81, 1)
I19 Sound of tapping (pen, foot, finger)	2.2 (1.3)	2 (1–3)	0.95	0.81	0.86 (0.82, 1)
I20 Keyboard tapping	1.5 (1.3)	1 (0–3)	0.97	0.85	0.86 (0.82, 1)
I21 Rustling plastic or paper	1.5 (1.4)	1 (0–3)	0.96	0.80	0.85 (0.81, 1)
I22 Cutting nails	2.1 (1.4)	2 (1–3)	0.95	0.77	0.85 (0.81, 1)
I23 Chewing gum	3.4 (1)	4 (3–4)	0.97	0.82	0.83 (0.78, 1)
I24 Footsteps	0.8 (1.1)	0 (0–1)	0.95	0.66	0.77 (0.72, 1)
I25 Cutlery noises	2.3 (1.4)	2 (1–4)	0.94	0.76	0.84 (0.79, 1)
I26 Sneezing	0.8 (1.1)	0 (0–1)	0.96	0.70	0.83 (0.78, 1)
I27 Kissing	1.5 (1.4)	1 (0–3)	0.96	0.72	0.87 (0.82, 1)
I28 Joint cracking	1.2 (1.3)	1 (0–2)	0.97	0.82	0.83 (0.79, 1)
I29 Muffled	2.0 (1.4)	2 (1–3)	0.97	0.89	0.89 (0.85, 1)
I30 Baby crying	1.7 (1.4)	1 (0–3)	0.97	0.88	0.87 (0.83, 1)
I31 Repetitive barking	1.8 (1.3)	2 (1–3)	0.97	0.85	0.87 (0.83, 1)
I32 Car engine	0.5 (0.9)	0 (0–1)	0.95	0.50	0.76 (0.71, 1)
I33 Clock ticking	1.2 (1.3)	1 (0–2)	0.96	0.80	0.84 (0.79, 1)
I34 Humming of object	1.2 (1.2)	1 (0–2)	0.95	0.81	0.84 (0.81, 1)
I35 Bass sounds	1.4 (1.5)	1 (0–3)	0.92	0.70	0.82 (0.78, 1)

sd: standard deviation; Q1, Q3: first and third quartile; Psi: psi nonparametric coefficient of agreement.

**Table 2:** Classical test theory, factor analysis and item response theory parameters estimation for the binary items in the misophonia specific sample (N = 613).

Item	Trigger Endorsement	EFA Loadings (Promax Rotation)			Internal Consistency *		2-PL IRT Model Parameters	
	N (%)	F1	F2	F3	AID	ITC	a (sd)	b (sd)
I2 mouth	549 (89.9)	1.03			0.75	0.60	4.3 (0.9)	−1.4 (0.1)
I6 smacking	438 (71.5)	0.75			0.75	0.61	1.6 (0.2)	−0.8 (0.1)
I1 eating	279 (45.6)	0.73			0.74	0.69	2.3 (0.3)	0.1 (0.1)
I4 mushy	268 (43.8)	0.72			0.74	0.67	2.1 (0.3)	0.2 (0.1)
I5 swallowing	209 (34.3)	0.66	0.31		0.75	0.64	2.2 (0.3)	0.5 (0.1)
I3 crunching	396 (64.6)	0.64			0.75	0.64	1.6 (0.2)	−0.5 (0.1)
I7 slurping	421 (68.7)	0.64	0.25		0.74	0.66	1.8 (0.2)	−0.7 (0.1)
I23 gum	548 (89.4)	0.58			0.77	0.45	1.3 (0.2)	−2.1 (0.2)
I9 blocked nose	361 (59.1)		0.69		0.48	0.61	1.8 (0.4)	−0.3 (0.1)
I8 breathing	62 (10.1)		0.63		0.54	0.39	1.7 (0.4)	1.9 (0.3)
I12 sniffing	455 (74.3)		0.57		0.52	0.53	1.1 (0.2)	−1.2 (0.2)
I11 coughing	397 (65.0)		0.45		0.51	0.56	0.9 (0.2)	−0.8 (0.2)
I10 throat	390 (63.8)		0.40		0.53	0.52	0.7 (0.2)	−0.9 (0.2)
I14 snoring	447 (73.0)		0.33	0.25	0.53	0.50	1.0 (0.2)	−1.2 (0.2)
I13 hiccups	269 (44.0)		0.31	0.27	0.53	0.53	0.7 (0.1)	0.4 (0.1)
I32 car	126 (20.7)			0.63	0.73	0.44	1.3 (0.2)	1.3 (0.2)
I35 bass	269 (44.0)			0.59	0.72	0.49	1.1 (0.1)	0.3 (0.1)
I19 tapping	380 (62.1)			0.56	0.72	0.48	1.1 (0.1)	−0.5 (0.1)
I15 letter	229 (37.4)			0.55	0.72	0.47	1.1 (0.2)	0.6 (0.1)
I21 rustling	314 (51.5)			0.55	0.72	0.50	1.2 (0.2)	−0.1 (0.1)
I16 accents	199 (32.6)			0.55	0.73	0.44	1.0 (0.1)	0.9 (0.1)
I17 words	207 (33.8)			0.49	0.73	0.41	0.9 (0.1)	0.9 (0.1)
I34 humming	271 (44.4)			0.49	0.73	0.44	0.9 (0.1)	0.3 (0.1)
I24 footsteps	213 (34.9)			0.46	0.73	0.45	1.0 (0.1)	0.8 (0.1)
I18 whistling	342 (55.9)			0.44	0.72	0.47	1.1 (0.1)	−0.3 (0.1)
I28 joint	278 (45.6)			0.43	0.73	0.41	0.8 (0.1)	0.3 (0.1)
I20 keyboard	323 (53.0)			0.39	0.73	0.39	0.8 (0.1)	−0.2 (0.1)
I29 muffled	369 (60.3)			0.38	0.73	0.39	0.8 (0.1)	−0.6 (0.1)
I33 clock	267 (43.6)			0.36	0.73	0.37	0.6 (0.1)	0.4 (0.2)
I27 kissing	347 (56.9)			0.36	0.74	0.33	0.5 (0.1)	−0.5 (0.2)
I26 sneezing	222 (36.3)		0.27	0.34	0.73	0.42	0.9 (0.1)	0.8 (0.1)
I30 crying	349 (57.1)			0.34	0.74	0.34	0.5 (0.1)	−0.6 (0.2)
I22 nails	381 (62.4)			0.29	0.73	0.37	0.6 (0.1)	−0.9 (0.2)
I31 barking	346 (56.4)			0.26	0.74	0.33	0.5 (0.1)	−0.5 (0.2)
I25 cutlery	420 (68.6)			0.21	0.74	0.29	0.5 (0.1)	−1.7 (0.4)

* alpha per factor: F1 = 0.77, F2 = 0.56, F3 = 0.74; EFA: exploratory factor analysis; 2-PL: 2-parameter logistic; IRT: item response theory; F1–F3: factors 1–3; AID: alpha if item deleted; ITC: item-total correlations; a: discrimination parameter; b: difficulty parameter; sd: standard deviation.

**Table 3:** Odds ratios of trigger endorsement in relation to misophonia (adjusted for age, gender, depression and generalised anxiety reported diagnoses).

Trigger Sound	Dimension	Odds Ratio	Unadjusted *p*-Value	B&H *p*-Value
I1 Normal eating	EaS	42.9	**<0.001**	**<0.001**
I2 Chewing/Mouth open	EaS	43.6	**<0.001**	**<0.001**
I3 Crunching	EaS	107.5	**<0.001**	**<0.001**
I4 Mushy foods	EaS	18.8	**<0.001**	**<0.001**
I5 Swallowing	EaS	15.1	**0.010**	**0.027**
I6 Lip smacking	EaS	28.1	**<0.001**	**<0.001**
I7 Slurping	EaS	23.0	**<0.001**	**<0.001**
I8 Normal breathing	N/TS	-	**-**	**-**
I9 Loud/unusual breathing (blocked nose)	N/TS	21.8	**<0.001**	**<0.001**
I10 Throat clearing	N/TS	1.0	0.905	0.994
I11 Repetitive coughing	N/TS	1.8	0.107	0.208
I12 Repetitive sniffing	N/TS	3.4	**0.002**	**0.008**
I13 Hiccups	N/TS	1.0	0.983	0.994
I14 Snoring	N/TS	2.4	**0.026**	0.061
I15 Certain letter sounds	GES	2.6	0.113	0.208
I16 Certain accents	GES	1.0	0.952	0.994
I17 Certain words	GES	1.7	0.200	0.333
I18 Whistling sound	GES	2.9	**0.007**	**0.022**
I19 Sound of tapping (pen, foot, finger)	GES	1.2	0.708	0.918
I20 Keyboard tapping	GES	2.6	**0.013**	**0.032**
I21 Rustling plastic or paper	GES	3.0	**0.005**	**0.018**
I22 Cutting nails	GES	2.7	**0.009**	**0.026**
I23 Chewing gum	EaS	12.1	**<0.001**	**<0.001**
I24 Footsteps	GES	1.2	0.618	0.865
I25 Cutlery noises	GES	3.5	**0.002**	**0.008**
I26 Sneezing	GES	0.7	0.334	0.531
I27 Kissing	GES	1.3	0.444	0.648
I28 Joint cracking	GES	0.9	0.859	0.994
I29 Muffled	GES	1.9	0.086	0.177
I30 Baby crying	GES	1.1	0.901	0.994
I31 Repetitive barking	GES	1.6	0.185	0.324
I32 Car engine	GES	0.9	0.823	0.994
I33 Clock ticking	GES	1.4	0.421	0.641
I34 Humming of object	GES	1.2	0.673	0.906
I35 Bass sounds	GES	2.4	**0.031**	0.068

Bold *p*-values indicate statistically significant odds ratios; Eating sounds were coded as EaS, nose/throat sounds were coded as N/TS and general environment sounds were coded as GES. B&H: Benjamini–Hochberg post hoc adjustment for multiple comparisons.

## Data Availability

The data presented in this study are available on request from the corresponding author.
